# Cardiovascular Phenotypes Across Major Cancer Types: Distribution and Association with All-Cause Mortality in a Retrospective Cohort Study

**DOI:** 10.3390/diagnostics16152342

**Published:** 2026-07-27

**Authors:** Bozhidar Krastev, Natalia Spasova, Desislava Somleva, Angelina Borizanova, George Dimitrov, Elena Kinova, Assen Goudev

**Affiliations:** 1 Department of Emergency Medicine, Medical University of Sofia, 1527 Sofia, Bulgaria; spasova.natalia@gmail.com (N.S.); desisomleva@abv.bg (D.S.); borizanowa@abv.bg (A.B.); kinova.e@abv.bg (E.K.); agoudev@abv.bg (A.G.); 2 Clinic of Cardiology, University Hospital “Tsaritsa Yoanna-ISUL”, 1527 Sofia, Bulgaria; 3 Clinic of Medical Oncology, Medical University of Sofia, University Hospital “Tsaritsa Yoanna”, 1527 Sofia, Bulgaria; gdimitrov@medfac.mu-sofia.bg

**Keywords:** oncology patients, cardiovascular phenotypes, cardio-oncology, comorbidity, recorded all-cause mortality

## Abstract

**Background/Objectives**: Cardiovascular comorbidities are frequent in patients with solid malignancies, but their distribution across tumor types and their association with recorded all-cause mortality remain insufficiently defined. This study assessed clinically defined cardiovascular phenotypes across major cancer groups and examined their association with recorded all-cause mortality. **Methods**: We performed a retrospective cohort study of 2020 consecutive adult patients hospitalized with malignancies at University Hospital “Tsaritsa Yoanna—ISUL” between September 2023 and December 2025. Patients were classified as having respiratory, gastrointestinal, genitourinary, breast, or other solid malignancies. Four non-mutually exclusive cardiovascular phenotypes were predefined: atherosclerotic, cardiometabolic, structural heart disease, and thromboembolic. Mortality status was ascertained through the National Health Insurance Fund database and hospital electronic records. As complete time-to-event data were unavailable, mortality was analyzed as a binary outcome using multivariable logistic regression. **Results**: Recorded all-cause mortality occurred in 471 patients (23.3%) and differed significantly across solid tumor types (*p* < 0.001). Mortality was highest in respiratory cancers (42.3%), followed by gastrointestinal cancers (30.2%), other malignancies (23.5%), genitourinary cancers (14.9%), and breast cancer (9.1%). Cardiovascular phenotypes also differed across tumor groups. In the fully adjusted model, structural heart disease was associated with recorded all-cause mortality status after multivariable adjustment (OR 1.61, 95% CI 1.16–2.23, *p* = 0.004). The atherosclerotic phenotype showed a borderline association (OR 1.42, 95% CI 1.00–2.02, *p* = 0.051), whereas cardiometabolic and thromboembolic phenotypes were not associated with recorded mortality after multivariable adjustment. Model AUC was 0.771. When cardiovascular burden was modeled continuously, each additional cardiovascular factor was associated with higher odds of recorded all-cause mortality status (OR 1.12, 95% CI 1.03–1.21, *p* = 0.005). **Conclusions**: Cardiovascular profiles differed across cancer types. Structural heart disease was the most consistent cardiovascular phenotype associated with recorded all-cause mortality, while overall cardiovascular burden provided additional but modest mortality-related information. In our cohort, routinely recorded cardiovascular diagnoses helped characterize mortality-related clinical profiles. However, these findings should be viewed as observational and hypothesis-generating, and further longitudinal studies are needed before any clinical use can be recommended.

## 1. Introduction

According to the World Health Organization, the global burden of cancer continues to increase, with more than 20 million new cases registered each year [1]. Together with cardiovascular disease, cancer represents one of the leading causes of morbidity and mortality worldwide. The coexistence of neoplastic disease and cardiovascular pathology is therefore frequently observed and may be explained by shared risk factors such as advanced age, obesity, smoking, diabetes, and hormone replacement therapy, which are involved both in the pathogenesis of cardiovascular disease and in carcinogenesis [2,3,4].

With improvements in early diagnosis and advances in oncological treatment, including targeted therapy and immunotherapy, patient outcomes have improved, leading to a growing population of cancer survivors [5,6]. In this setting, cardiovascular comorbidities have become increasingly relevant. They may worsen clinical outcomes, limit therapeutic options, and identify patients at higher risk of adverse cardiovascular events [7,8,9]. Although survival is primarily determined by tumor type and stage, the presence of significant comorbidity has also been associated with higher overall mortality in several oncological populations [10,11,12,13]. Despite this, most studies have focused on individual comorbid conditions or isolated cardiovascular risk factors. Less is known about whether routinely available cardiovascular diagnoses can be grouped into simple, clinically interpretable phenotypes across different cancer types. It also remains unclear whether such predefined cardiovascular phenotypes provide mortality-related information beyond tumor type, stage, and metastatic disease. Therefore, the present study aimed to assess the distribution of predefined cardiovascular phenotypes across major solid tumor groups and to evaluate their association with recorded all-cause mortality after adjustment for demographic and oncologic variables.

## 2. Materials and Methods

### 2.1. Study Design and Population

The present study is a retrospective observational cohort study including 2020 consecutive adult patients hospitalized in the Oncology Clinic of University Hospital “Tsaritsa Yoanna—ISUL”, Sofia, Bulgaria, between September 2023 and December 2025. Patients with solid malignancies were categorized according to primary tumor site as respiratory, gastrointestinal, genitourinary, breast, or other solid cancers. Demographic characteristics, oncologic variables, cardiovascular comorbidities, and cardiovascular risk factors were extracted from electronic medical records. Cancer stage (I–IV) and metastatic disease status were also recorded and included in the analyses as separate clinicopathologic variables.

### 2.2. Outcome Definition

The primary outcome was recorded all-cause mortality at the time of data extraction. Mortality status was ascertained through the National Health Insurance Fund database and hospital electronic records. Exact dates of death and complete follow-up duration were unavailable for all patients. Consequently, survival analyses could not be performed and mortality was analyzed as a binary outcome using logistic regression. The reported odds ratios should therefore be interpreted as associations with recorded mortality status rather than estimates of time-dependent mortality risk.

Because patients entered the cohort at different calendar times, the potential observation period from hospitalization to data extraction was not uniform. This may have introduced follow-up bias, as patients hospitalized earlier had a longer opportunity for death to occur and be recorded than those hospitalized later. Therefore, the present analyses should be interpreted strictly as associations with recorded all-cause mortality status at data extraction, rather than as estimates of survival, prognosis, or time-dependent mortality risk.

### 2.3. Cardiovascular Phenotypes

Cardiovascular phenotypes were predefined a priori as clinically relevant, non-mutually exclusive categories, allowing individual patients to be assigned to more than one phenotype.

The following cardiovascular phenotypes were used:

- Atherosclerotic phenotype: coronary artery disease, prior myocardial infarction, peripheral/vascular disease, and prior stroke in the absence of atrial fibrillation.
- Cardiometabolic phenotype: arterial hypertension, diabetes mellitus, obesity, and dyslipidemia.
- Structural heart disease phenotype: atrial fibrillation, valvular heart disease, and heart failure.
- Thromboembolic phenotype: deep vein thrombosis and/or pulmonary embolism, including asymptomatic pulmonary embolism.

The atherosclerotic phenotype was defined by the presence of at least one atherosclerotic component. To reduce potential misclassification of cardioembolic stroke as atherosclerotic disease, prior stroke was included in this phenotype only when atrial fibrillation was absent.

The cardiometabolic phenotype was defined by the presence of at least two cardiometabolic components, reflecting clustering of metabolic and vascular risk factors. The structural heart disease phenotype and thromboembolic phenotype were defined by the presence of at least one component, as these conditions are clinically relevant even when present in isolation.

Each phenotype was coded as a binary variable. A sensitivity analysis was performed to assess the impact of stroke classification on the atherosclerotic phenotype. Alternative definitions included: (1) inclusion of all stroke cases, regardless of atrial fibrillation status, and (2) exclusion of stroke from the atherosclerotic phenotype. The results of these sensitivity analyses are presented in Supplementary Table S1. The overlap between cardiovascular phenotypes is summarized in Supplementary Table S2.

These cardiovascular phenotypes were investigator-defined pragmatic clinical groupings and were not intended to represent validated phenotype classifications or data-driven clusters. They were created to group routinely available cardiovascular diagnoses and risk factors into clinically interpretable domains. Atherosclerotic, structural heart disease, and thromboembolic phenotypes were defined by at least one component because each included condition is clinically relevant when present alone. The cardiometabolic phenotype required at least two components in order to reflect clustering of metabolic risk factors rather than an isolated common risk factor.

### 2.4. Cardiovascular Burden

In addition to phenotype-based classification, a cumulative cardiovascular burden score was calculated based on the total number of cardiovascular comorbidities and risk factors present in each patient. Patients were categorized into three groups according to cardiovascular burden: 0–1 factors, 2–3 factors, and ≥4 factors. The association between cardiovascular burden and recorded all-cause mortality was assessed using logistic regression.

### 2.5. Statistical Analysis

Statistical analyses were performed using SPSS software, version 23.0 (IBM Corp., Armonk, NY, USA).

Continuous variables were tested for normality using the One-Sample Kolmogorov–Smirnov test. Normally distributed variables are presented as mean ± standard deviation, whereas non-normally distributed variables are expressed as median and interquartile range. Categorical variables are presented as counts and percentages. Missing data were handled using an available-case approach for descriptive analyses. No imputation was performed. The variables included in the main multivariable logistic regression models were available for the full cohort; therefore, the primary models were fitted using all 2020 patients.

Comparisons among tumor-type groups were performed using the Kruskal–Wallis test for continuous variables and Pearson’s chi-square test for categorical variables. Data are presented as *n* (%), with percentages calculated within tumor-type groups.

Multivariable binary logistic regression analysis was performed to assess associations between demographic, oncologic, and cardiovascular variables and recorded all-cause mortality status. The clinical-oncologic model included age, sex, tumor type, cancer stage, and metastatic disease. A second model additionally included the four cardiovascular phenotypes. Results are presented as odds ratios with 95% confidence intervals. Exploratory interaction analyses were performed by adding tumor type × cardiovascular phenotype interaction terms separately to the fully adjusted model. A global interaction test was also performed.

Model performance was assessed using both discrimination and calibration. Discrimination was evaluated using the area under the receiver operating characteristic curve. Nested model comparison was performed to assess whether adding cardiovascular phenotypes changed model fit and discrimination for recorded all-cause mortality status beyond demographic and oncologic variables, using change in AUC and likelihood-ratio testing. Calibration was assessed using the Hosmer–Lemeshow goodness-of-fit test and by comparing observed and predicted recorded mortality across deciles of predicted probability. Multicollinearity was assessed using variance inflation factors, and the actual VIF values are reported in the Supplementary Material. Internal validation of the full multivariable logistic regression model was performed using bootstrap resampling to estimate potential model optimism. A total of 1000 bootstrap resamples were generated. For each resample, the model was refitted and discrimination was assessed in both the bootstrap sample and the original dataset. Mean optimism was calculated as the average difference between bootstrap-sample AUC and original-dataset AUC. The optimism-corrected AUC was then obtained by subtracting mean optimism from the apparent AUC. Additional exploratory incremental performance analyses were performed to further assess the effect of adding cardiovascular phenotypes to the clinical-oncologic model. These included integrated discrimination improvement, continuous and categorical net reclassification improvement, and decision curve analysis. Categorical net reclassification improvement was calculated using predicted-probability categories of <10%, 10–20%, 20–30%, and ≥30%. Decision curve analysis was performed across selected clinically plausible threshold probabilities.

Although cancer stage and metastatic disease are clinically related variables, both were retained in the model because no relevant multicollinearity was detected and they captured distinct information in the available dataset.

A two-sided *p*-value < 0.05 was considered statistically significant.

Because multiple univariable comparisons were performed across tumor-type groups, no formal adjustment for multiplicity was applied. These comparisons were considered descriptive and exploratory; therefore, their *p*-values should be interpreted as hypothesis-generating rather than confirmatory.

### 2.6. Ethics

The study was conducted in accordance with the Declaration of Helsinki and was approved by the institutional ethics committee of University Hospital “Tsaritsa Yoanna—ISUL”, Sofia, Bulgaria. Due to the retrospective nature of the study, the requirement for informed consent was waived.

## 3. Results

### 3.1. Characteristics of the Study Population

A total of 2020 patients were included in the analysis. Patients were stratified according to tumor type: respiratory cancers *n* = 350, gastrointestinal cancers *n* = 530, genitourinary cancers *n* = 509, breast cancer *n* = 427, and other malignancies *n* = 204.

Age differed significantly across tumor types (*p* < 0.001). Patients with genitourinary and gastrointestinal cancers were older, with median ages of 70 years (IQR 62–75) and 68 years (IQR 61–74), respectively. Patients with respiratory cancers, breast cancer, and other malignancies were younger, with median ages of 62 years (IQR 56–69), 63 years (IQR 52–72), and 62 years (IQR 52–70), respectively.

Sex distribution also differed across groups (*p* < 0.001). Male sex was most frequent among patients with respiratory cancers (282/350, 80.6%) and genitourinary cancers (355/509, 69.7%), whereas breast cancer patients were almost exclusively female, with only 8 male patients (1.9%).

Cancer stage distribution varied markedly by tumor type (*p* < 0.001). Stage IV disease was most common in respiratory cancers (210/350, 60.0%), followed by other malignancies (68/204, 33.3%) and gastrointestinal cancers (171/530, 32.3%). In contrast, stage IV disease was less frequent in genitourinary cancers (94/509, 18.5%) and breast cancer (51/427, 11.9%).

Metastatic disease was present in 388 patients (19.2%) and differed significantly across tumor types (*p* < 0.001). The highest proportion was observed in gastrointestinal cancers (143/530, 27.0%), followed by respiratory cancers (74/350, 21.1%), genitourinary cancers (91/509, 17.9%), other malignancies (28/204, 13.7%), and breast cancer (52/427, 12.2%).

Recorded all-cause mortality occurred in 471 patients (23.3%). Mortality differed substantially across tumor types (*p* < 0.001). It was highest in respiratory cancers (148/350, 42.3%), followed by gastrointestinal cancers (160/530, 30.2%), other malignancies (48/204, 23.5%), genitourinary cancers (76/509, 14.9%), and breast cancer (39/427, 9.1%) (Table 1).

### 3.2. Cardiovascular Comorbidities, Risk Factors and Thromboembolic Events

Cardiovascular comorbidities and risk factors were unevenly distributed across tumor types (Table 2).

Atherosclerotic conditions were more common in genitourinary and respiratory cancers. Coronary artery disease was most frequent in genitourinary cancers (45/509, 8.8%), followed by respiratory cancers (23/350, 6.6%) and gastrointestinal cancers (26/530, 4.9%) (*p* < 0.001). Prior myocardial infarction followed a similar pattern and was most frequent in genitourinary cancers (29/509, 5.7%) (*p* < 0.001). Prior stroke was observed most often in respiratory cancers (26/350, 7.4%) and genitourinary cancers (32/509, 6.3%) (*p* < 0.001).

Heart failure was present in 112 patients and differed between tumor groups (*p* = 0.004). The highest rates were observed in gastrointestinal cancers (40/530, 7.5%) and respiratory cancers (26/350, 7.4%), whereas the lowest rates were found in breast cancer (13/427, 3.0%) and other malignancies (5/204, 2.5%).

Atrial fibrillation also differed significantly across groups (*p* = 0.003). It was most frequent in gastrointestinal cancers (61/530, 11.5%), followed by genitourinary cancers (48/509, 9.4%) and respiratory cancers (27/350, 7.7%). Vascular disease and valvular disease did not differ significantly across tumor types (*p* = 0.123 and *p* = 0.114, respectively).

Cardiometabolic risk factors showed clear variation. Diabetes mellitus was most frequent in gastrointestinal cancers (103/530, 19.4%), followed by genitourinary cancers (84/509, 16.5%) (*p* < 0.001). Hypertension was most common in genitourinary cancers (345/509, 67.8%), followed by gastrointestinal cancers (321/530, 60.6%) (*p* < 0.001). Obesity was most frequent in breast cancer (134/427, 31.4%) and genitourinary cancers (158/509, 31.0%) (*p* < 0.001). Dyslipidemia was also more frequent in genitourinary cancers (49/509, 9.6%) (*p* = 0.006).

Smoking was most common in respiratory cancers (173/350, 49.4%), followed by other malignancies (57/204, 27.9%) and gastrointestinal cancers (129/530, 24.3%) (*p* < 0.001). Alcohol use was highest in respiratory cancers (55/350, 15.7%) and gastrointestinal cancers (77/530, 14.5%) (*p* < 0.001).

Combined thromboembolic events, including pulmonary embolism, asymptomatic pulmonary embolism and deep vein thrombosis, were present in 110 patients (5.4%). Their frequency differed across tumor types (*p* = 0.005), with the highest rate in gastrointestinal cancers (46/530, 8.7%). Rates were lower in respiratory cancers (16/350, 4.6%), other malignancies (9/204, 4.4%), genitourinary cancers (22/509, 4.3%), and breast cancer (17/427, 4.0%).

### 3.3. Cardiovascular Phenotypes According to Tumor Type

Cardiovascular phenotypes were defined as non-mutually exclusive categories. The primary atherosclerotic phenotype included coronary artery disease, prior myocardial infarction, vascular disease, and prior stroke only in the absence of atrial fibrillation. The cardiometabolic phenotype required at least two components among diabetes mellitus, hypertension, obesity, and dyslipidemia. Structural heart disease and thromboembolic phenotypes were defined by the presence of at least one component (Table 3).

Phenotypes were defined as non-mutually exclusive categories; therefore, a single patient could belong to more than one phenotype.

All four cardiovascular phenotypes differed significantly across tumor types (Table 4).

The atherosclerotic phenotype was present most often in genitourinary cancers (86/509, 16.9%) and respiratory cancers (53/350, 15.1%). Lower rates were observed in gastrointestinal cancers (46/530, 8.7%), other malignancies (13/204, 6.4%), and breast cancer (18/427, 4.2%) (*p* < 0.001).

The cardiometabolic phenotype was most frequent in genitourinary cancers (187/509, 36.7%), followed by gastrointestinal cancers (157/530, 29.6%) and breast cancer (115/427, 26.9%). Lower rates were found in respiratory cancers (62/350, 17.7%) and other malignancies (35/204, 17.2%) (*p* < 0.001).

The structural heart disease phenotype was most frequent in gastrointestinal cancers (89/530, 16.8%), followed by genitourinary cancers (71/509, 13.9%) and respiratory cancers (43/350, 12.3%). It was less common in breast cancer (31/427, 7.3%) and other malignancies (12/204, 5.9%) (*p* < 0.001).

The thromboembolic phenotype was most frequent in gastrointestinal cancers (46/530, 8.7%). Rates in the remaining groups were lower and broadly similar: respiratory cancers (16/350, 4.6%), other malignancies (9/204, 4.4%), genitourinary cancers (22/509, 4.3%), and breast cancer (17/427, 4.0%) (*p* = 0.005).

### 3.4. Multivariable Analysis for Recorded All-Cause Mortality

Multivariable binary logistic regression was performed to identify factors associated with recorded all-cause mortality (Table 5). The model included age, sex, tumor type, cancer stage, metastatic disease, and the four cardiovascular phenotypes.

Increasing age was associated with higher odds of recorded all-cause mortality status (OR 1.02 per year, 95% CI 1.01–1.03, *p* = 0.001). Male sex was also associated with increased mortality (OR 1.36, 95% CI 1.03–1.78, *p* = 0.029).

Using breast cancer as the reference group, respiratory cancer was associated with the highest adjusted odds of recorded all-cause mortality status (OR 3.64, 95% CI 2.25–5.89, *p* < 0.001). Gastrointestinal cancers were also associated with higher mortality (OR 2.44, 95% CI 1.59–3.75, *p* < 0.001), as were other malignancies (OR 2.31, 95% CI 1.37–3.88, *p* = 0.002). Compared with breast cancer, genitourinary cancer was not associated with recorded mortality after multivariable adjustment (OR 1.13, 95% CI 0.70–1.85, *p* = 0.611). Among cancer stage categories, stage IV disease remained independently associated with mortality (OR 3.36, 95% CI 2.08–5.42, *p* < 0.001). Stage II and stage III disease were not statistically significant compared with stage I. Metastatic disease was independently associated with mortality (OR 2.10, 95% CI 1.49–2.96, *p* < 0.001).

Among cardiovascular phenotypes, structural heart disease was associated with recorded all-cause mortality after multivariable adjustment (OR 1.61, 95% CI 1.16–2.23, *p* = 0.004). The atherosclerotic phenotype showed a borderline association (OR 1.42, 95% CI 1.00–2.02, *p* = 0.051). The cardiometabolic phenotype (OR 0.95, 95% CI 0.73–1.24, *p* = 0.699) and the thromboembolic phenotype (OR 1.01, 95% CI 0.63–1.62, *p* = 0.965) were not associated with recorded mortality after multivariable adjustment. The full model included 2020 patients and 471 recorded deaths and yielded an AUC of 0.771 for recorded all-cause mortality status. Because all variables included in the primary multivariable model were available for the full cohort, no patients were excluded from this model because of missing covariate data.

Multicollinearity was low in the full multivariable model. All variance inflation factor values were below 5, with the highest value observed for stage IV versus stage I disease (VIF = 3.40). Full VIF values are shown in Supplementary Table S3.

Exploratory interaction analyses were performed to assess whether the association between cardiovascular phenotypes and recorded all-cause mortality differed by tumor type. No statistically significant interaction was observed for the atherosclerotic phenotype (LR χ^2^ = 4.86, df = 4, *p* = 0.302), cardiometabolic phenotype (LR χ^2^ = 3.30, df = 4, *p* = 0.508), structural heart disease phenotype (LR χ^2^ = 4.90, df = 4, *p* = 0.298), or thromboembolic phenotype (LR χ^2^ = 3.74, df = 4, *p* = 0.442). When all interaction terms were entered together, the global interaction test was also not significant (LR χ^2^ = 18.11, df = 16, *p* = 0.318). These analyses were considered exploratory because some tumor type–phenotype combinations included relatively small numbers of patients.

### 3.5. Model Fit After Adding Cardiovascular Phenotypes

A clinical-oncologic model including age, sex, tumor type, cancer stage and metastatic disease achieved an AUC of 0.767. After adding the four cardiovascular phenotypes, the AUC increased to 0.771 (Table 6).

Bootstrap internal validation showed limited optimism in model discrimination. The apparent AUC of the full model was 0.771, the mean bootstrap optimism was 0.0087, and the optimism-corrected AUC was 0.763. No bootstrap samples failed to converge.

The clinical-oncologic model had an AUC of 0.767. After adding the four cardiovascular phenotypes, the AUC increased only slightly to 0.771, corresponding to an absolute change of +0.004. Although the likelihood-ratio test showed a statistically significant improvement in model fit (χ^2^ = 13.17, df = 4, *p* = 0.010), the improvement in discrimination was very small. Therefore, this finding should be interpreted as modest additional model information for recorded all-cause mortality status, rather than as a clinically meaningful improvement in model discrimination. Calibration of the full model was acceptable, with no evidence of poor fit on the Hosmer–Lemeshow test (χ^2^ = 7.91, df = 8, *p* = 0.443).

The calibration plot comparing observed and predicted recorded mortality across deciles of predicted probability is presented in Figure S1.

Exploratory incremental performance analyses were consistent with this cautious interpretation. The integrated discrimination improvement was small (IDI = 0.0076), while the continuous net reclassification improvement was modest and not statistically robust (continuous NRI = 0.090, 95% CI −0.103 to 0.238). The categorical NRI was minimal (0.015). Decision curve analysis showed only small and inconsistent differences in net benefit between the two models across the evaluated threshold probabilities. These results are presented in Supplementary Table S4 and support the interpretation that cardiovascular phenotypes provided limited additional model information rather than clinically meaningful improvement in discrimination, reclassification, or net clinical benefit.

### 3.6. Cumulative Cardiovascular Burden and Recorded All-Cause Mortality

In an exploratory analysis, recorded all-cause mortality increased across categories of cumulative cardiovascular burden (Table 7), from 19.4% in patients with 0–1 cardiovascular factors to 26.4% in those with 2–3 factors and 30.3% in those with ≥4 factors. In the adjusted categorical model, the 2–3 factor group remained significantly associated with mortality compared with the 0–1 factor group (adjusted OR 1.31, 95% CI 1.02–1.68, *p* = 0.036), whereas the ≥4 factor group did not reach statistical significance (adjusted OR 1.30, 95% CI 0.90–1.88, *p* = 0.155).

This finding should not be interpreted as evidence of a threshold effect; rather, the lack of statistical significance in the ≥4-factor category likely reflects limited precision and reduced statistical power in this smaller subgroup, despite the highest observed mortality rate. When modeled continuously, each additional cardiovascular factor was associated with higher odds of recorded all-cause mortality status (adjusted OR 1.12, 95% CI 1.03–1.21, *p* = 0.005).

### 3.7. Sensitivity Analysis of the Atherosclerotic Phenotype

Sensitivity analyses were performed to assess whether the association between the atherosclerotic phenotype and mortality depended on the handling of prior stroke (Supplementary Table S1).

In the primary definition, stroke was counted as part of the atherosclerotic phenotype only when atrial fibrillation was absent. With this definition, the atherosclerotic phenotype showed a borderline association with mortality OR 1.42, 95% CI 1.00–2.02, *p* = 0.051. When all stroke cases were included regardless of atrial fibrillation status, the result remained similar (OR 1.39, 95% CI 0.98–1.96, *p* = 0.065). When stroke was excluded entirely, the association weakened (OR 1.20, 95% CI 0.80–1.78, *p* = 0.377).

In contrast, the structural heart disease phenotype remained associated with recorded mortality after multivariable adjustment across all sensitivity models, with ORs ranging from 1.59 to 1.64 and *p*-values between 0.003 and 0.006.

### 3.8. Overlap of Cardiovascular Phenotypes

Cardiovascular phenotypes were not mutually exclusive (Supplementary Table S2). Overall, 1182 patients (58.5%) had no cardiovascular phenotype, 596 patients (29.5%) had one phenotype, 197 patients (9.8%) had two phenotypes, 42 patients (2.1%) had three phenotypes, and 3 patients (0.1%) had all four phenotypes.

This overlap was addressed using both phenotype-based analyses and the cumulative cardiovascular burden approach.

## 4. Discussion

In this cohort of 2020 oncology patients, cardiovascular comorbidities and predefined cardiovascular phenotypes differed substantially across tumor types. Mortality was highest in respiratory and gastrointestinal cancers and lowest in breast cancer. After adjustment for demographic and oncologic variables, structural heart disease showed the most consistent statistical association with recorded all-cause mortality among the predefined cardiovascular phenotypes. The atherosclerotic phenotype showed a borderline association, whereas cardiometabolic and thromboembolic phenotypes were not associated with mortality after multivariable adjustment. Exploratory interaction analyses did not suggest that the associations between cardiovascular phenotypes and recorded mortality differed by tumor type. However, these findings should be interpreted cautiously because some tumor type–phenotype combinations included relatively few patients. An important observation from our cohort is that cardiovascular comorbidity was not uniform across tumor types, and its association with mortality differed according to the phenotype considered.

This is relevant because cancer and cardiovascular disease often coexist through shared risk factors, ageing, inflammation, and treatment-related mechanisms. Previous studies have suggested links between heart failure after myocardial infarction and subsequent cancer [14], possible bidirectional interactions between cardiovascular injury and tumor biology [15], associations between atherosclerotic cardiovascular disease risk and cancer incidence [16], and inflammatory links between cancer and cardiovascular disease [17].

The distribution of cardiovascular disease in the present cohort was heterogeneous. It is noteworthy that the male sex predominated across all cancer groups, with the exception of breast cancer. This may partly reflect differences in risk-factor exposure and tumor-site distribution, a finding that has also been observed in other studies [18].

It should be noted that the studied population also has a significant cardiovascular burden, a finding that has been reported in other studies as well [19].

Our findings are consistent with previous retrospective data showing that cardiovascular comorbidity varies by tumor site [20].

Thromboembolic events were most frequent in gastrointestinal cancers, which is clinically plausible given the known association between gastrointestinal malignancies and venous thromboembolism [21]. However, the thromboembolic phenotype was not associated with recorded mortality after multivariable adjustment. This may be because thromboembolic events are strongly linked to cancer type, stage, and metastatic disease, variables that were already included in the multivariable model. In addition, the relatively low number of thromboembolic events may have limited the precision of this analysis. In this cohort, thromboembolic events described part of the cardiovascular profile, but there was no evidence of an additional adjusted association with recorded mortality.

The structural heart disease phenotype was the most consistent cardiovascular phenotype associated with recorded all-cause mortality. This finding should not be interpreted as evidence that structural heart disease is the strongest biological determinant of mortality, as residual confounding, the composite nature of the phenotype, and the heterogeneous observation period may have influenced the observed association. A possible explanation is that heart failure, atrial fibrillation, and valvular heart disease reflect established cardiac pathology rather than isolated risk-factor exposure. These conditions may reduce cardiovascular reserve, increase vulnerability to anemia, infection, inflammation, fluid shifts, and cancer-related systemic stress, and may also limit tolerance to oncologic treatment. Therefore, the observed association may reflect a combination of impaired cardiac reserve, frailty, and competing non-cancer morbidity rather than a direct causal effect of structural heart disease itself.

This may partly explain why structural heart disease showed a more consistent adjusted association than the cardiometabolic phenotype in this dataset.

The composite nature of these cardiovascular phenotypes should also be considered when interpreting the results. Each phenotype grouped conditions that are clinically related but not biologically identical. For example, the atherosclerotic phenotype included coronary artery disease, prior myocardial infarction, peripheral vascular disease, and ischemic stroke without atrial fibrillation. Although these conditions share an atherosclerotic background, they may differ in severity, timing, treatment, and clinical consequences. Similarly, the structural heart disease phenotype combined atrial fibrillation, heart failure, and valvular heart disease, which may influence recorded mortality through different mechanisms, including impaired cardiac reserve, arrhythmia burden, frailty, or treatment limitations. Therefore, the observed associations should be interpreted as composite-level associations and should not be assumed to reflect the effect of any single component.

The atherosclerotic phenotype showed only a borderline association with mortality. This result should be interpreted cautiously, as it represents a weak and definition-dependent association. The sensitivity analysis showed that the association depended partly on how prior stroke was classified. This is important because stroke mechanisms are heterogeneous, and some events may be cardioembolic rather than atherosclerotic, particularly in patients with atrial fibrillation.

The cardiometabolic phenotype was common but was not associated with recorded mortality after multivariable adjustment. Hypertension, diabetes, obesity, and dyslipidemia are important long-term cardiovascular risk factors, but in a hospitalized oncology cohort, their adjusted association with recorded mortality may be attenuated by tumor type, stage, metastatic disease, and established cardiac disease. Recent multinational cohort data also showed that pre-existing cardiovascular disease and type 2 diabetes, either separately or combined, are associated with higher all-cause and cause-specific mortality in adults with cancer, supporting the clinical relevance of cardiometabolic comorbidity in oncology populations [22].

Phenotype-based classification has become increasingly relevant in cardiovascular medicine, including hypertension, sleep apnea, cardiac imaging populations, and other cardiovascular settings [23,24,25].

Li and colleagues showed that cardiovascular risk among cancer survivors varies by cancer type, cardiovascular outcome, age, and sex. Although not described as phenotypes, these differences support the concept of clinically heterogeneous cardiovascular risk in oncology populations [26]. This is also consistent with Mitchell et al., who reported that cardiovascular risk profiles and cardiovascular event rates differ across cancer types, supporting the need to consider tumor site when interpreting cardio-oncology risk [27].

More recent population-based data also showed that long-term cardiovascular risk after systemic cancer treatment differs according to cancer type and treatment context, further supporting the need for tumor-specific cardiovascular risk interpretation [8].

The present study applies a clinically defined phenotype approach to an unselected oncology cohort. The findings suggest that such classification may provide a pragmatic descriptive framework, while the mortality-related associations differed across phenotypes. Structural heart disease showed the most consistent adjusted association with recorded all-cause mortality status in this dataset, while the atherosclerotic phenotype was weaker and definition-dependent.

The model comparison also supports a cautious interpretation. Adding the four cardiovascular phenotypes to the clinical-oncologic model was associated with a statistically significant improvement in model fit, but the absolute increase in AUC was only +0.004. This indicates that the practical contribution of these phenotypes to model discrimination was very limited. Therefore, cardiovascular phenotypes should be viewed as providing modest additional model information related to recorded mortality status, rather than a meaningful improvement in model discrimination. Tumor-related factors remained the main contributors to model discrimination. Bootstrap internal validation suggested limited optimism in the full model; however, external validation in an independent cohort remains necessary before any broader clinical use of the model can be considered. The exploratory cumulative cardiovascular burden analysis gave a similar message. Mortality increased across burden categories, but the adjusted categorical associations were partly attenuated. When cardiovascular burden was analyzed as a continuous variable, each additional cardiovascular factor remained associated with recorded mortality after multivariable adjustment. This suggests that the overall cardiovascular profile may provide additional descriptive information on recorded mortality status; however, cardiovascular burden should be interpreted only as an additional mortality-related marker in this dataset. These findings fit with previous work showing the importance of cardiovascular risk assessment in cancer populations. Established cardiovascular risk scores have been associated with later cardiovascular events in breast cancer, and population-level data suggest that cardiovascular mortality is an important cause of death among cancer patients and survivors [28,29]. The present study extends this concept by showing that cardiovascular phenotype distributions and cumulative burden vary across tumor groups, while structural heart disease and cumulative cardiovascular burden showed adjusted associations with recorded all-cause mortality.

Several limitations should be acknowledged. The study was retrospective and single-center, which limits causal inference and external validity. A major methodological limitation is the absence of exact dates of death and complete follow-up duration. Because patients were hospitalized at different time points during the study period, their potential observation time until data extraction was not uniform. This may have introduced follow-up bias, since patients hospitalized earlier had a longer opportunity for death to occur and be recorded than patients hospitalized later. Consequently, mortality could only be analyzed as a binary recorded outcome, and survival analyses, hazard ratios, overall survival, progression-free survival, or time-dependent mortality estimates could not be calculated. The reported odds ratios should therefore be interpreted only as associations with recorded all-cause mortality status, not as prognostic or survival estimates. In addition, the composite cardiovascular phenotypes combined conditions with different biological mechanisms and clinical implications. Therefore, the study cannot determine which individual component within each phenotype contributed most strongly to the observed association with recorded mortality status. In addition, cause-of-death adjudication was not available, preventing differentiation between cardiovascular and cancer-related mortality. Detailed oncologic treatment exposures, including systemic anticancer therapy, radiation therapy, and potentially cardiotoxic treatments, were not systematically available. These factors may have influenced both cardiovascular phenotypes and recorded all-cause mortality, and their absence from the models may have contributed to residual confounding. Furthermore, phenotype definitions were based on routinely available clinical variables and administrative data and were not externally validated; therefore, some degree of misclassification is possible, particularly in the classification of cerebrovascular events. Finally, as with all observational designs, residual confounding from unmeasured or incompletely captured variables cannot be excluded. Finally, because numerous univariable comparisons were performed without formal multiplicity correction, the possibility of type I error cannot be excluded, and these findings should be interpreted as exploratory.

Overall, cardiovascular phenotype patterns differed across cancer types. Structural heart disease remained associated with recorded all-cause mortality after multivariable adjustment, whereas cardiometabolic and thromboembolic phenotypes did not. Atherosclerotic disease showed a borderline, definition-dependent association. The four cardiovascular phenotypes provided modest additional model information, but their practical contribution to discrimination was limited compared with tumor-related factors. Higher cumulative cardiovascular burden was separately associated with recorded all-cause mortality.

## 5. Conclusions

In this cohort, cardiovascular comorbidities and phenotype patterns differed substantially across tumor types, and recorded all-cause mortality increased with higher cumulative cardiovascular burden.

Among the predefined cardiovascular phenotypes, structural heart disease showed the most consistent adjusted association with recorded all-cause mortality. The atherosclerotic phenotype showed a borderline association that depended on the handling of prior stroke, while cardiometabolic and thromboembolic phenotypes were not associated with mortality after multivariable adjustment. These findings do not establish that cardiovascular phenotype-based classification improves clinical risk prediction or should directly guide patient management. Rather, they show that routinely available cardiovascular diagnoses may help describe mortality-related clinical profiles in hospitalized oncology patients. Further studies incorporating longitudinal follow-up, treatment exposure, exact dates of death, and cause-specific mortality are needed to determine whether such phenotype-based classification has meaningful value for clinical risk assessment in oncology populations.

## Figures and Tables

**Table 1:** Baseline oncologic characteristics according to tumor type.

Characteristic	Overall *n* = 2020	Respiratory *n* = 350	GI *n* = 530	Genitourinary *n* = 509	Breast *n* = 427	Other *n* = 204	*p*-Value
Age, years, median (IQR)	66 (58–73)	62 (56–69)	68 (61–74)	70 (62–75)	63 (52–72)	62 (52–70)	<0.001
Male sex, *n* (%)	1079 (53.4%)	282 (80.6%)	304 (57.4%)	355 (69.7%)	8 (1.9%)	130 (63.7%)	<0.001
Cancer stage, *n* (%)							<0.001
Stage I	396 (19.6%)	11 (3.1%)	37 (7.0%)	231 (45.4%)	89 (20.8%)	28 (13.7%)	
Stage II	458 (22.7%)	33 (9.4%)	113 (21.3%)	129 (25.3%)	133 (31.1%)	50 (24.5%)	
Stage III	572 (28.3%)	96 (27.4%)	209 (39.4%)	55 (10.8%)	154 (36.1%)	58 (28.4%)	
Stage IV	594 (29.4%)	210 (60.0%)	171 (32.3%)	94 (18.5%)	51 (11.9%)	68 (33.3%)	
Metastatic disease, *n* (%)	388 (19.2%)	74 (21.1%)	143 (27.0%)	91 (17.9%)	52 (12.2%)	28 (13.7%)	<0.001
Recorded all-cause mortality, *n* (%)	471 (23.3%)	148 (42.3%)	160 (30.2%)	76 (14.9%)	39 (9.1%)	48 (23.5%)	<0.001

Data are presented as median (interquartile range) or *n* (%) calculated within tumor-type groups. Percentages are calculated within the total cohort for the Overall column and within each tumor-type group for tumor-type columns. *p*-values compare the five tumor-type groups. The Kruskal–Wallis test was used for age, and the Pearson χ^2^ test was used for categorical variables.

**Table 2:** Cardiovascular comorbidities, risk factors and thromboembolic events according to tumor type.

Variable	Respiratory *n* = 350	GI *n* = 530	Genitourinary *n* = 509	Breast *n* = 427	Other *n* = 204	*p*-Value
CAD	23 (6.6%)	26 (4.9%)	45 (8.8%)	6 (1.4%)	3 (1.5%)	<0.001
MI	15 (4.3%)	19 (3.6%)	29 (5.7%)	4 (0.9%)	3 (1.5%)	<0.001
Stroke	26 (7.4%)	15 (2.8%)	32 (6.3%)	10 (2.3%)	2 (1.0%)	<0.001
Heart failure	26 (7.4%)	40 (7.5%)	28 (5.5%)	13 (3.0%)	5 (2.5%)	0.004
Atrial fibrillation	27 (7.7%)	61 (11.5%)	48 (9.4%)	24 (5.6%)	9 (4.4%)	0.003
Vascular disease	12 (3.4%)	15 (2.8%)	18 (3.5%)	4 (0.9%)	5 (2.5%)	0.123
Valvular disease	7 (2.0%)	20 (3.8%)	15 (2.9%)	7 (1.6%)	2 (1.0%)	0.114
Diabetes mellitus	33 (9.4%)	103 (19.4%)	84 (16.5%)	50 (11.7%)	23 (11.3%)	<0.001
Hypertension	172 (49.1%)	321 (60.6%)	345 (67.8%)	205 (48.0%)	88 (43.1%)	<0.001
Dyslipidemia	21 (6.0%)	30 (5.7%)	49 (9.6%)	17 (4.0%)	10 (4.9%)	0.006
Obesity	50 (14.3%)	115 (21.7%)	158 (31.0%)	134 (31.4%)	36 (17.6%)	<0.001
Smoking	173 (49.4%)	129 (24.3%)	103 (20.2%)	57 (13.3%)	57 (27.9%)	<0.001
Alcohol use	55 (15.7%)	77 (14.5%)	49 (9.6%)	13 (3.0%)	19 (9.3%)	<0.001
Thromboembolic events combined	16 (4.6%)	46 (8.7%)	22 (4.3%)	17 (4.0%)	9 (4.4%)	0.005

Data are presented as *n* (%), with percentages calculated within tumor-type groups. *p*-values compare the five tumor-type groups using the Pearson χ^2^ test.

**Table 3:** Definitions of cardiovascular phenotypes.

Phenotype	Definition
Atherosclerotic phenotype ≥ 1	CAD, MI, vascular disease, and stroke only in the absence of AF
Cardiometabolic phenotype ≥ 2	At least two of diabetes mellitus, hypertension, obesity, and dyslipidemia
Structural heart disease phenotype ≥ 1	HF, AF, or valvular disease
Thromboembolic phenotype ≥ 1	PE, including asymptomatic PE, or DVT

**Table 4:** Cardiovascular phenotypes according to tumor type.

Phenotype	Respiratory *n* = 350	GI *n* = 530	Genitourinary *n* = 509	Breast *n* = 427	Other *n* = 204	*p*-Value
Atherosclerotic phenotype ≥ 1	53 (15.1%)	46 (8.7%)	86 (16.9%)	18 (4.2%)	13 (6.4%)	<0.001
Cardiometabolic phenotype ≥ 2	62 (17.7%)	157 (29.6%)	187 (36.7%)	115 (26.9%)	35 (17.2%)	<0.001
Structural heart disease phenotype ≥ 1	43 (12.3%)	89 (16.8%)	71 (13.9%)	31 (7.3%)	12 (5.9%)	<0.001
Thromboembolic phenotype ≥ 1	16 (4.6%)	46 (8.7%)	22 (4.3%)	17 (4.0%)	9 (4.4%)	0.005

Data are presented as *n* (%), with percentages calculated within tumor-type groups. *p*-values compare the five tumor-type groups using the Pearson χ^2^ test.

**Table 5:** Multivariable logistic regression analysis for recorded all-cause mortality.

Variable	OR	95% CI	*p*-Value
Age, per year	1.02	1.01–1.03	0.001
Male sex	1.36	1.03–1.78	0.029
Tumor type			
Respiratory vs. breast cancer	3.64	2.25–5.89	<0.001
Gastrointestinal vs. breast cancer	2.44	1.59–3.75	<0.001
Genitourinary vs. breast cancer	1.13	0.70–1.85	0.611
Other vs. breast cancer	2.31	1.37–3.88	0.002
Cancer stage			
Stage II vs. stage I	1.33	0.84–2.10	0.219
Stage III vs. stage I	1.48	0.95–2.32	0.083
Stage IV vs. stage I	3.36	2.08–5.42	<0.001
Metastatic disease	2.10	1.49–2.96	<0.001
Atherosclerotic phenotype ≥ 1	1.42	1.00–2.02	0.051
Cardiometabolic phenotype ≥ 2	0.95	0.73–1.24	0.699
Structural heart disease phenotype ≥ 1	1.61	1.16–2.23	0.004
Thromboembolic phenotype ≥ 1	1.01	0.63–1.62	0.965

Model includes age, sex, tumor type, cancer stage, metastatic disease, and four cardiovascular phenotypes. Reference categories: female sex, breast cancer, stage I, no metastases, and absence of each phenotype. Model *n* = 2020, deaths = 471; AUC = 0.771.

**Table 6:** Model fit, discrimination, and calibration.

Model	AUC	ΔAUC	LR χ^2^	df	LR *p*-Value	HL χ^2^	df	HL *p*-Value
Model 1	0.767	Reference	—	—	—	11.14	8	0.194
Model 2	0.771	+0.004	13.17	4	0.010	7.91	8	0.443

AUC, area under the receiver operating characteristic curve; LR, likelihood-ratio; HL, Hosmer–Lemeshow. Model 1 included age, sex, tumor type, cancer stage, and metastatic disease. Model 2 additionally included the four cardiovascular phenotypes.

**Table 7:** Adjusted association between cumulative cardiovascular burden and recorded all-cause mortality.

Cardiovascular Burden	Alive, *n* (%)	Dead, *n* (%)	Total, *n*	Mortality (%)	Adjusted OR	95% CI	*p*-Value
0–1 factors	821 (80.6)	198 (19.4)	1019	19.4	1.00	Reference	-
2–3 factors	567 (73.6)	203 (26.4)	770	26.4	1.31	1.02–1.68	0.036
≥4 factors	161 (69.7)	70 (30.3)	231	30.3	1.30	0.90–1.88	0.155
Cardiovascular burden, per 1-factor increase	-	-	-	-	1.12	1.03–1.21	0.005

Data are presented as *n* (%), unless otherwise indicated. Adjusted odds ratios were calculated using binary logistic regression and adjusted for age, sex, tumor type, cancer stage, and metastatic disease. Cardiovascular burden was calculated as the number of cardiovascular comorbidities/risk factors listed in Table 2, with thromboembolic events counted as one combined component. The categorical and continuous cardiovascular burden models were fitted separately. The 0–1 factors category was used as the reference group. OR, odds ratio; CI, confidence interval.

## Data Availability

The datasets used and analyzed during the current study are available from the corresponding author on reasonable request.

## Supplementary Materials

The following supporting information can be downloaded at https://www.mdpi.com/article/10.3390/diagnostics16152342/s1. Table S1. Sensitivity analysis of the atherosclerotic phenotype definition; Table S2. Overlap of cardiovascular phenotypes; Table S3. Variance inflation factor values; Table S4. Exploratory incremental performance metrics after adding cardiovascular phenotypes; Figure S1. Calibration plot of the full multivariable logistic regression model.

## Abbreviations

The following abbreviations are used in this manuscript:

AF	Atrial fibrillation
AUC	Area under the receiver operating characteristic curve
CAD	Coronary artery disease
CI	Confidence interval
DVT	Deep vein thrombosis
GI	Gastrointestinal
HF	Heart failure
HL	Hosmer–Lemeshow
IDI	Integrated discrimination improvement
IQR	Interquartile range
LR	Likelihood-ratio
MI	Myocardial infarction
NRI	Net reclassification improvement
OR	Odds ratio
PE	Pulmonary embolism
VIF	Variance inflation factor
