# Supplementary material for: Cardiovascular Phenotypes Across Major Cancer Types: Distribution and Association with All-Cause Mortality in a Retrospective Cohort Study
Source: Diagnostics (Basel). 2026 Jul 27;16(15):2342. doi: 10.3390/diagnostics16152342 (PMC13464043; doi:10.3390/diagnostics16152342)
Supplement: Supplementary file 1 [file diagnostics-16-02342-s001.zip › Supplementary Figure S1.pdf]

Figure S1. Calibration plot of the full multivariable logistic regression model

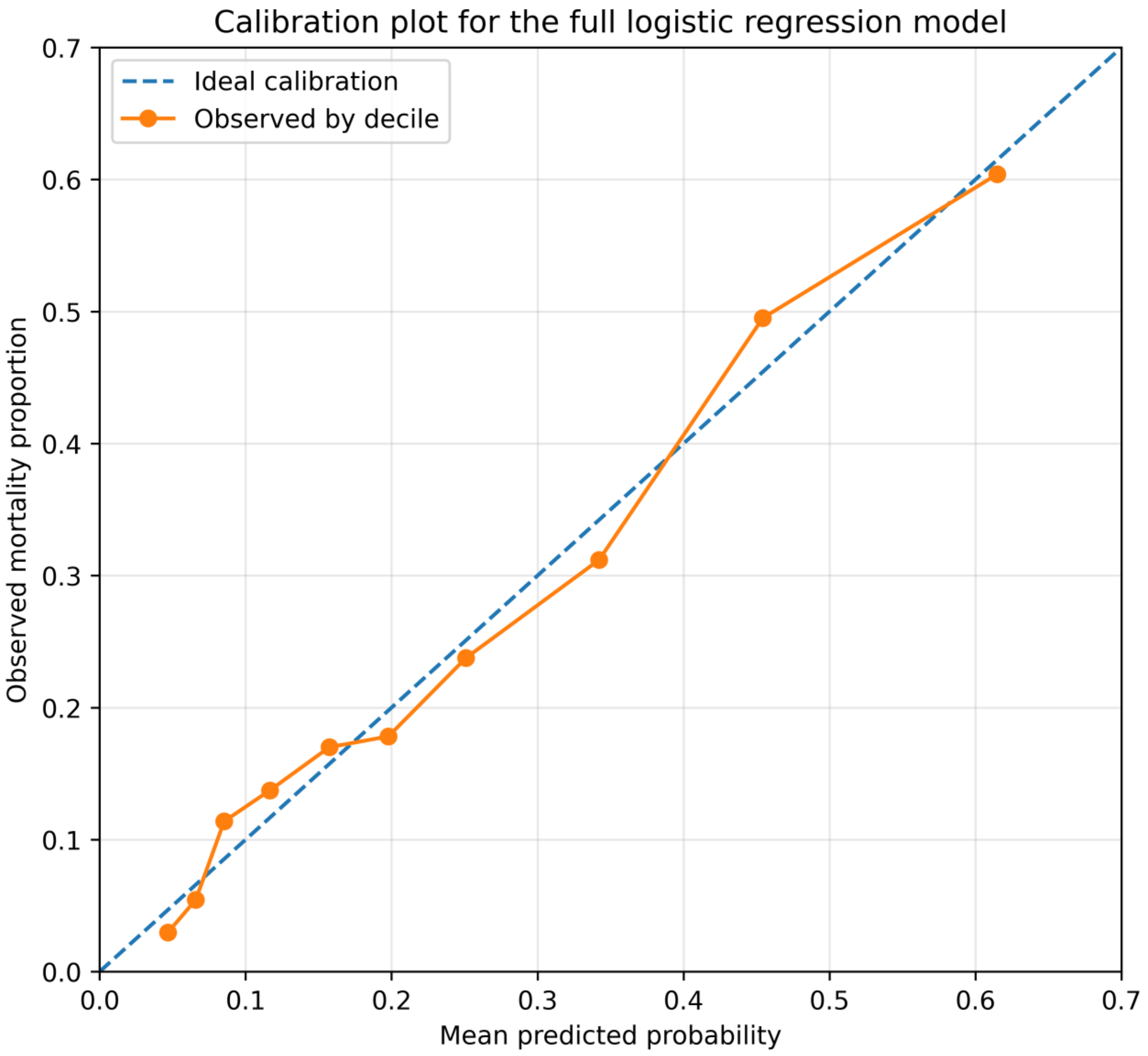

Observed and predicted recorded all-cause mortality across deciles of predicted probability. The model included age, sex, tumor type, cancer stage, metastatic disease, and the four predefined cardiovascular phenotypes.

Calibration table for the full model

Decile	n	Observed deaths	Expected deaths	Observed mortality (%)	Mean predicted probability (%)
1	202	6	9.4	3.0	4.7
2	202	11	13.3	5.4	6.6
3	202	23	17.2	11.4	8.5
4	204	28	23.8	13.7	11.7

5	200	34	31.5	17.0	15.7
6	202	36	40.0	17.8	19.8
7	202	48	50.7	23.8	25.1
8	202	63	69.1	31.2	34.2
9	202	100	91.8	49.5	45.4
10	202	122	124.2	60.4	61.5

Observed and expected recorded mortality are shown by deciles of predicted probability from the full model.
