# Supplementary material for: Cardiovascular Phenotypes Across Major Cancer Types: Distribution and Association with All-Cause Mortality in a Retrospective Cohort Study
Source: Diagnostics (Basel). 2026 Jul 27;16(15):2342. doi: 10.3390/diagnostics16152342 (PMC13464043; doi:10.3390/diagnostics16152342)
Supplement: Supplementary file 1 [file diagnostics-16-02342-s001.zip › Supplementary Table S1 and S2.pdf]

Supplementary Table S1. Sensitivity analysis of the atherosclerotic phenotype definition

Atherosclerotic phenotype definition	Atherosclerotic OR	95% CI	p-value	Structural OR	95% CI	p-value	AUC
Primary: stroke counted only if AF absent	1.42	1.00–2.02	0.051	1.61	1.16–2.23	0.004	0.771
Sensitivity 1: all stroke cases included	1.39	0.98–1.96	0.065	1.59	1.14–2.20	0.006	0.771
Sensitivity 2: stroke excluded entirely	1.20	0.80–1.78	0.377	1.64	1.18–2.27	0.003	0.770

All sensitivity models included age, sex, tumor type, cancer stage, metastatic disease, cardiometabolic phenotype, structural phenotype, thromboembolic phenotype, and the respective atherosclerotic phenotype definition.

Supplementary Table S2. Overlap of cardiovascular phenotypes

Number of phenotypes per patient	n	%
0	1182	58.5%
1	596	29.5%
2	197	9.8%
3	42	2.1%
4	3	0.1%

Overlap was assessed using the primary phenotype definitions. Cardiovascular phenotypes were non-mutually exclusive.
