# Supplementary material for: Cardiovascular Phenotypes Across Major Cancer Types: Distribution and Association with All-Cause Mortality in a Retrospective Cohort Study
Source: Diagnostics (Basel). 2026 Jul 27;16(15):2342. doi: 10.3390/diagnostics16152342 (PMC13464043; doi:10.3390/diagnostics16152342)
Supplement: Supplementary file 1 [file diagnostics-16-02342-s001.zip › Supplementary Table S3 and S4.pdf]

Supplementary Table S3. Variance inflation factor values

Variable	VIF
Age	1.17
Male sex	1.49
Respiratory vs breast cancer	2.30
Gastrointestinal vs breast cancer	2.09
Genitourinary vs breast cancer	2.35
Other vs breast cancer	1.58
Stage 2 vs stage I	1.79
Stage 3 vs stage I	2.11
Stage 4 vs stage I	3.40
Metastatic disease	1.99
Atherosclerotic phenotype	1.10
Cardiometabolic phenotype	1.08
Structural heart disease phenotype	1.09
Thromboembolic phenotype	1.02

All VIF values were below 5, indicating no relevant multicollinearity among the variables included in the multivariable model.

Supplementary Table S4. Exploratory incremental performance metrics after adding cardiovascular phenotypes.

Panel A. Reclassification and discrimination metrics

Metric	Value	95% CI	Interpretation
IDI	0.0076	0.0029 to 0.0198	Small improvement in average separation between patients with and without recorded mortality.
Continuous NRI	0.090	-0.103 to 0.238	Modest and not statistically robust.
Categorical NRI	0.015	Not calculated	Very small reclassification gain; risk categories were exploratory.

Panel B. Decision curve analysis across selected threshold probabilities

Threshold probability	Model 1 net benefit	Model 2 net benefit	Difference (Model 2 - Model 1)
10%	0.1602	0.1598	-0.0004
20%	0.1045	0.1063	+0.0018
25%	0.0851	0.0903	+0.0052
30%	0.0731	0.0728	-0.0003
40%	0.0452	0.0492	+0.0040

Model 1 included age, sex, tumor type, cancer stage, and metastatic disease. Model 2 additionally included the four cardiovascular phenotypes. The categorical NRI used exploratory risk categories of <10%, 10-20%, 20-30%, and >=30% predicted probability. CI, confidence interval; IDI, integrated discrimination improvement; NRI, net reclassification improvement.
